# Gender specific differences in the liver proteome of rats exposed to short term and low-concentration hexabromocyclododecane (HBCD)†
†Electronic supplementary information (ESI) available. See DOI: 10.1039/c6tx00166a


**DOI:** 10.1039/c6tx00166a

**Published:** 2016-06-30

**Authors:** I. Miller, C. Diepenbroek, E. Rijntjes, J. Renaut, K. J. Teerds, C. Kwadijk, S. Cambier, A. J. Murk, A. C. Gutleb, T. Serchi

**Affiliations:** a Institute for Medical Biochemistry , Department for Biomedical Sciences , University of Veterinary Medicine Vienna , Veterinaerplatz 1 , A-1210 Vienna , Austria . Email: ingrid.miller@vetmeduni.ac.at; b Wageningen University , Human and Animal Physiology , P.O. Box 338 , 6700 AH Wageningen , The Netherlands; c Charité-Universitätsmedizin Berlin , Institute of Experimental Endocrinology , Augustenburger Platz 1 , 13353, Berlin , Germany; d Environmental Research and Innovation (ERIN) Department , Luxembourg Institute of Science and Technology (LIST) , 5 , avenue des Hauts-Fourneaux , L-4362 Esch-sur-Alzette , Grand-duchy of Luxembourg . Email: tommaso.serchi@list.lu ; Tel: +352-470 261; e Wageningen Institute for Marine Resources & Ecosystem Studies , IMARES , IJmuiden , The Netherlands; f Wageningen University , Marine Animal Ecology Group , De Elst 1 , 6708 WD Wageningen , The Netherlands

## Abstract

The influence of short term (7-day) exposure of male rats to the brominated flame retardant hexabromocyclododecane (HBCD) was studied by investigation of the liver proteome, both in euthyroid and hypothyroid rats and by comparing results with general data on animal physiology and thyroid hormone, leptin, insulin and gonadotropin concentrations determined in parallel. Proteome analysis of liver tissue by two-dimensional fluorescence difference gel electrophoresis (2D-DIGE) revealed that only small protein pattern changes were induced by exposure in males, on just a few proteins with different functions and not involved in pathways in common. This is in contrast to previous findings in similarly exposed eu- and hypothyroid female rats, where general metabolic pathways had been shown to be affected. The largest gender-dependent effects concerned basal concentrations of liver proteins already in control and hypothyroid animals, involving mainly the pathways which were also differently affected by HBCD exposure. Among them were differences in lipid metabolism, which – upon exposure to HBCD – may also be the reason for the considerably higher ratio of γ-HBCD accumulated in white adipose tissue of exposed female rats compared to males. The results further elucidate the already suggested different sensitivity of genders towards HBCD exposure on the protein level, and confirm the need for undertaking toxicological animal experiments in both genders.

## Abstract 2

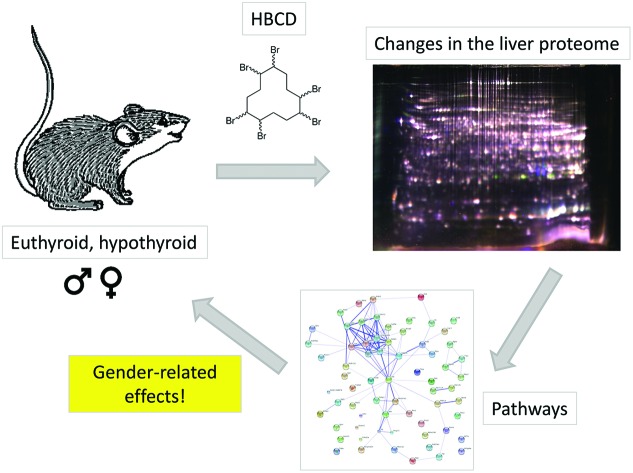
Gender specific impact of HBCD on rat liver proteome, determined by 2D-DIGE.

## Introduction

Since 2013, the brominated flame retardant hexabromocyclododecane (HBCD) is banned in most European countries, but outside Europe still being produced and widely used (*e.g.* in the US). HBCD mainly serves as an additive in building industry (in polystyrene foams), but is also used for impregnation in equipment. Due to its persistent nature, HBCD is expected to remain present in food chains for several years to come. Like other substances of this chemical class, HBCD is lipophilic and accumulates in adipose tissue, not only in experimental animals but also in humans.^1^–^5^ While animal studies have shown low acute toxicity of HBCD, there are toxicological effects, especially in liver, on the immune system and on serum thyroid hormone (TH) dependent developmental processes as well as indications of endocrine disrupting properties.^6^–^10^ In cell culture, recently also an enhancing effect on cell migration and invasion has been described for this compound.^11^ Further elucidation of the underlying mechanisms of HBCD action would be needed for a better understanding of its adverse effects, and specific investigations applying modern analytical methods are likely to help in this task.

Previous studies with 28d exposure of rats and variable doses of HBCD (between 0 and 200 mg per kg bw per day) revealed dose- and gender-dependent effects on body and organ weights as well as on TH levels, suggesting that female rats were more sensitive to exposure than male rats.^8^ In a similar experimental setup, transcriptomic data of rat liver and specific investigation of cytochrome P450 family members (CYPs) indicated gender-specific differences concerning affected genes/proteins related to metabolic pathways.^6^,^7^ Chronic exposure over longer intervals of time is likely to cause also secondary effects, which are likewise detected when screening for overall gene or protein level changes. Therefore, short term exposure may result in a different data set, better revealing primary effects of exposure to HBCD. Similarly, proteomic investigations are preferable to transcriptomic studies, as they show the actual protein concentrations in the organs under investigation.^12^,^13^

A recent HBCD short term (7 day) exposure study investigated the changes in the liver proteome of female rats and found a number of proteins differentially regulated in abundance, mainly related to general metabolic processes.^10^ In the present study, this investigation is extended to male rats and the experiment was performed under identical setup and conditions. This included euthyroid and hypothyroid animals as well as different HBCD exposure groups. Influences of dose, thyroid status and gender (including data from the previous experiment) on changes of the liver proteome are compared and further related to general animal data and hormone status determined in parallel. In the following, animal groups were named with an additional “m” and “f” depending on the gender (male, female) for easier comparison.

## Animals, material and methods

### Animals, treatment and experimental protocol

The animal experiment, incl. housing conditions and all experimental procedures, was approved under number 2006-051 by the Animal Welfare Committee of Wageningen University.

The animal experiment was performed as the one described in detail,^10^ except that for the present investigation male euthyroid and hypothyroid rats were used instead of females. Hypothyroidism was induced during foetal life by feeding the dams an iodide-poor diet based on AIN 1993 requirements, supplemented with 0.75% sodium perchlorate to deplete endogenous iodide stores, as described in detail.^14^ After weaning the offspring continued on the same diet as the dams until the experimental intervention. In brief, 86-day-old male healthy euthyroid controls (mET) or hypothyroid (mHT) rats were fed 0, 3 or 30 mg of HBCD per kg body weight added to commercial custard pudding for 7 days. Group size was 6 animals (6 groups). At the termination of the experiment, blood was collected by heart puncture (5 IU heparin per ml blood), and animals were sacrificed by decapitation. Liver and white adipose tissues were collected, snap frozen and stored at –80 °C, plasma was prepared and stored at –20 °C.

### Hormone measurements

Concentrations of total and free triiodothyronine (T_3_), corticosterone, leptin, luteinizing hormone (LH), follicle-stimulating hormone (FSH), and thyroid stimulating hormone (TSH) were determined by radioimmunoassays (RIAs), as previously described.^10^,^14^ In addition, RIAs for total thyroxine (T_4_) (Diagnostic Systems Laboratories, Texas, USA) (DSL-3200), testosterone (DSL-4100), and insulin (Linco Research, St. Charles, USA) were performed according to the manufacturers’ protocols. The detection limits of these assays were: 5 ng ml^–1^ for total T_4_, 0.1 ng ml^–1^ for testosterone and 0.5 ng ml^–1^ for insulin. Intra- and interassay variations were determined using several pools of rat sera and were less than 10%.

### Determination of internal HBCD dose

Chemical analyses to determine the internal levels of HBCD were performed at IMARES, The Netherlands. In brief, adipose tissue samples spiked with ^13^C-labeled HBCD as internal standards. Samples were then extracted and α-, β- and γ-diastereomeres were determined by LC-ESI-MS as described.^10^

### Statistical analysis (for hormonal measurements)

Data is expressed as mean ± standard deviation (SD). Statistical analysis was carried out using SPSS 19.0 for Windows. Data was tested for normality using the Shapiro-Wilk test. If normality could not be assumed, data were LOG10 transformed. Group means were compared using a two-way Univariate Analysis of Variance (ANOVA). Correlation between internal γ-HBCD dose and other parameters was tested with the Spearman's rank correlation test. Values of *P* < 0.05 were considered to be significant.

### Proteomics

For the proteomic experiments, four liver samples per experimental group (mET and mHT; 0, 3 and 30 mg per kg per day HBCD, respectively) were randomly selected, subjected to two-dimensional fluorescence difference gel electrophoresis (2D-DIGE) and further evaluated as previously described.^10^ In brief, rat livers were homogenized in lysis buffer (urea 7 M; thiourea 2 M; CHAPS 2% w/w; tris 30 mM, containing protease inhibitor) and proteins in the supernatant after centrifugation minimally labelled with CyDyes. Separation was performed on 24 cm IPGs of a non-linear 3–10 pH-range followed by SDS-PAGE in 12.5% gels, and images were captured on a Typhoon 9400 (DeCyder 7.0 software package, GE Healthcare, Diegem, Belgium). Gels were matched and subjected to univariate and multivariate analysis in order to determine differentially regulated spots with following criteria: fold change of at least 1.3 and *P*-value <0.05.^10^,^15^ These spots were automatically picked, tryptically digested and underwent MALDI-TOF/TOF analysis. Protein identification was performed by searching spectra against the SwissProt database with *Rattus norvegicus* as taxonomy, usually one PMF and up to 8 MS/MS spectra per spot. Parameters for the search were set as follow: up to two missed cleavages allowed, 100 ppm tolerance in PMF, 0.75 Da mass tolerance for precursor ion mass, carbamidomethyl cysteine as fixed modification, oxidation of methionine and oxidation of tryptophan (single oxidation, double oxidation and kynurenin) as variable modifications. Identifications were considered to be significant when the combined MOWSE score had *P* < 0.05. Criteria for positive MS identifications were in accordance with the Paris Report (; http://www.mcponline.org/misc/ParisReport_Final.dtl).

Statistics, including univariate analysis (ANOVA and *t*-test) and multivariate analysis (two-way ANOVA), was performed using the Extended Data Analysis (EDA) module, which is present inside the Decyder 7.0 software package.

Additional information on proteins (localization, function) and pathway analysis were retrieved from publicly available databases: UniProtKB database (http://www.uniprot.org/), STRING: functional protein association networks, v 9.1 (; http://string-db.org/),^16^,^17^ for *Rattus norvegicus* proteins or genes, respectively, and for proteins with accession numbers from UniProt.

## Results and discussion

### Male rats

#### Basic animal data and hormone measurements

The mHT rats had significantly higher serum TSH and significantly lower free T_3_, total T_3_ and total T_4_ concentrations than mET rats (for all *P* < 0.001), which is in accordance with earlier work^14^ (Table 1). HBCD did not induce any significant changes in total T_4_ and T_3_ concentrations, only free T_3_ concentrations showed a mild HBCD effect in males (*P* = 0.044). The Spearman's correlation for free T_3_ and HBCD, however, was not significant. The already increased corticosterone concentrations of mHT tended to increase further with HBCD exposure (*P* = 0.056). Serum leptin concentrations were not further influenced by HBCD exposure, but only by thyroid status (*P* = 0.006) (in line with the literature report^18^). Insulin secretion capacity is known to be reduced in the offspring of hypothyroid dams, in the current experiment, however, the rats were not fasted prior to sacrifice, so this effect could not be reliably determined (measured insulin concentrations were significantly lower in HT animals, *P* < 0.001). The internal HBCD concentration in the white adipose tissue, however, was negatively correlated to insulin (Rho = –0.613, *P* < 0.01) in the hypothyroid rats (Table 1). The HBCD-related alterations of insulin and partly also leptin levels may indicate an influence of HBCD on glucose and lipid metabolism.^19^

**Table 1:** Animal data (males): animals were exposed to 0, 3, or 30 mg per kg bw per d for 7 days

	HBCD-group	0	3	30	Thyroid effect (*P*-value)	HBCD effect (*P*-value)	Interaction (*P*-value)	Spearman's Rho
Body weight (g)	ET	427 ± 36	428 ± 35	430 ± 27	<0.001	0.828	0.736	–0.045
	HT	165 ± 22	153 ± 45	146 ± 32				–0.272
Liver weight (g)	ET	16.30 ± 1.12	16.14 ± 1.93	16.44 ± 1.68	<0.001	0.867	0.766	0.058
	HT	5.49 ± 0.80	5.12 ± 1.71	4.78 ± 1.11				–0.295
TSH (ng ml^–1^)	ET	0.38 ± 0.34	1.27 ± 0.92	0.49 ± 0.27	<0.001	0.810	0.409	0.168
	HT	17.01 ± 5.06	14.51 ± 4.31	16.76 ± 4.40				–0.012
Total T4 (μg dl^–1^)	ET	4.4 ± 0.3	3.9 ± 0.8	4.2 ± 0.5	<0.001	0.189	0.229	–0.306
	HT	<lod	<lod	<lod				0.433
Total T3 (ng ml^–1^)	ET	230 ± 48	234 ± 41	196 ± 9	<0.001	0.189	0.587	–0.35
	HT	165 ± 12	177 ± 25	161 ± 47				–0.148
Free T3 (pg ml^–1^)	ET	5.95 ± 0.51	6.30 ± 0.61	5.27 ± 0.87	<0.001	0.044	0.326	–0.341
	HT	1.56 ± 0.31	2.39 ± 0.78	1.80 ± 0.60				0.041
LH (ng ml^–1^)	ET	0.61 ± 0.15	1.22 ± 0.51	0.88 ± 0.30	0.002	0.021	0.088	0.33
	HT	0.54 ± 0.18	0.62 ± 0.28	0.55 ± 0.13				0.018
FSH (ng ml^–1^)	ET	3.81 ± 0.43	5.04 ± 0.80	4.00 ± 0.83	0.065	0.039	0.491	0.161
	HT	3.72 ± 1.15	4.16 ± 1.75	2.91 ± 0.83				–0.359
Testosterone (ng ml^–1^)	ET	2.23 ± 1.93	1.09 ± 0.36	1.24 ± 0.42	0.645	0.751	0.217	–0.308
	HT	0.98 ± 0.65	1.35 ± 1.45	1.64 ± 1.54				0.233
Leptin (ng ml^–1^)	ET	8 ± 2	7 ± 2	8 ± 2	0.006	0.205	0.134	0.022
	HT	13 ± 4	12 ± 4	8 ± 4				–0.428
Insulin (ng ml^–1^)	ET	6.02 ± 1.92	5.18 ± 2.69	8.81 ± 3.77	<0.001	0.174	0.035	0.261
	HT	4.38 ± 2.08	2.26 ± 1.48	2.09 ± 0.81				–0.613**
Corticosterone (ng ml^–1^)	ET	149 ± 71	103 ± 62	222 ± 69	0.027	0.056	0.549	0.35
	HT	183 ± 46	242 ± 113	353 ± 260				0.193
Internal γ-HBCD (mg per kg lw)	ET	<lod	25.5 ± 5.2	81.0 ± 33.1	0.002	<0.001	0.013	
	HT	<lod	40.7 ± 8.6	130.0 ± 32.9				

Body weights and liver weights (given in Table 1) were both considerably smaller in mHT animals (similar to the literature report^14^), but did not differ between HBCD treated and non-treated groups of the same thyroid status.

Overall, the observed changes upon exposure to HBCD in mET animals were consistent with earlier reported HBCD effects on TH levels in the 28d study by van der Ven^8^ with ET rats. No studies have been reported with mHT rats.

#### HBCD adipose tissue concentrations

The α- and β-HBCD concentrations in the white adipose tissue were below the lower limit of quantification for all groups. γ-HBCD was detected in HBCD treated rats, and was significantly higher in mHT animals compared with mET animals (by about 60%). This cannot be explained by the smaller body weight of the HT animals, as the HBCD dose to which animals were exposed was related to the body weight. Accumulated γ-HBCD content was about 2 to 3 times higher in both 30 mg per kg per day groups compared to the respective 3 mg per kg per day groups (Table 1). γ-HBCD was the dominating stereoisomer in adipose tissue, similar to what was observed in a 28d study performed in ET animals.^8^ Both *in vitro* as well as *in vivo* research has shown that γ-HBCD can interfere with TH-functioning in the presence of TH, but not without.^20^,^21^

#### Proteomics

**Fig. 1:**
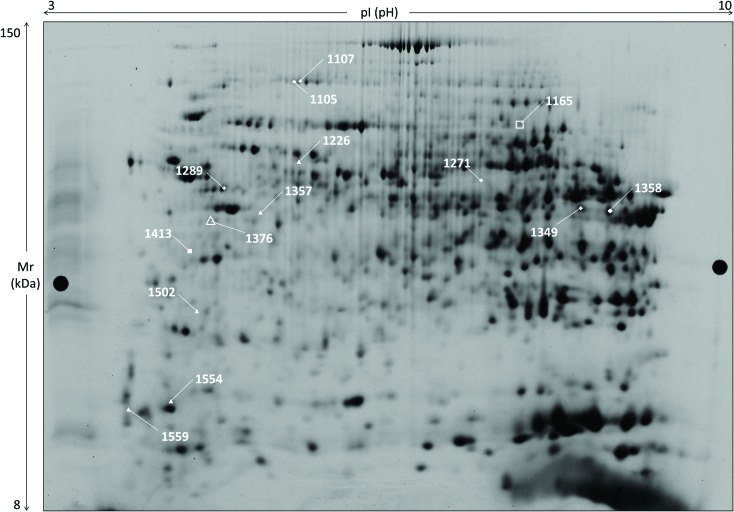
Two-dimensional gel image of a rat liver sample: 2D-DIGE separation of rat liver sample (master gel, grey level image). Marked spots show statistically significant changes in abundance upon HBCD exposure: 2 in mET animals (white squares), 6 in mHT rats (white triangles). In addition, 8 spots are of different abundance in ET and HT males (6 white diamonds + spots #1165, #1376 with other symbols). Detailed data on identifications is compiled in ESI Table 2.†

Two-dimensional electrophoresis of liver proteins gave complex patterns of about 3000 spots per gel (Fig. 1) and the evaluation included 24 gels of male rats’ samples of different exposure and thyroid state. Only a few spots were of significantly different abundance due to HBCD exposure or thyroid state (Fig. 1, Table 2), and were subjected to MS analyses (ESI Table 2;† 22).

**Table 2:** Comparison of HBCD-treated male rats

Significant changes											
(a) Effect of HBCD in ET-animals											
Spot number	Protein name	UniProt ID	mET3/mET0		mET30/mET0		mET30/mET3		Keywords/GO	Keywords/GO	Keywords/GO
			Av. Ratio	*T*-test	Av. Ratio	*T*-test	Av. Ratio	*T*-test	Biological process	Cellular component	Molecular function
1165	rCG56002		0.877	0.548	1.310	0.135	1.490	0.0189			
1413	Small glutamine-rich tetratricopeptide repeat-containing protein alpha	SGTA_RAT	0.980	0.897	1.270	0.0527	1.300	0.00644	Binds directly to HSC70 and HSP70 and	Cytoplasm	Chaperone

(b) Effect of HBCD in HT-animals											
Spot number	Protein name	UniProt ID	mHT3/mHT0		mHT30/mHT0		mHT30/mHT3		Keywords/GO	Keywords/GO	Keywords/GO
			Av. Ratio	*T*-test	Av. Ratio	*T*-test	Av. Ratio	*T*-test	Biological process	Cellular component	Molecular function
1226	Formimidoyltransferase-cyclodeaminase	FTCD_RAT	0.676	0.00852	0.800	0.0378	1.180	0.0753	His metabolism	Cytoplasm, cytoskeleton, golgi	Folic acid binding, His catabolic process
1357	Serum paraoxonase/arylesterase 2	PON2_RAT	0.877	0.193	1.340	0.144	1.530	0.0436	Aromatic compound catabolic process	Membrane	Arylesterase activity
1376	Serum paraoxonase/arylesterase 1	PON1_RAT	1.050	0.619	1.660	0.0501	1.580	0.0387	Multiple regulation/response	Secreted, HDL	Hydrolase
1502	Guanidinoacetate N-methyltransferase	GAMT_RAT	1.340	0.292	1.380	0.0477	1.030	0.708	Biosynthetic/metabolic processes		Methyltransferase, transferase
1554	Cytochrome b5	CYB5_RAT	0.658	0.0411	0.877	0.512	1.330	0.22	Electron transport, transport	ER, membrane, microsome	Metal ion binding electron carrier activity
1559	Calmodulin	CALM_RAT	1.560	0.041	1.390	0.341	0.885	0.588	Multitude of regulations	Cytoplasm, cytoskeleton	Modulation by Ca++

(c) Effect of TH status (same HBCD treatment)											
Spot number	Protein name	UniProt ID	mHT0/mET0		mHT3/mET3		mHT30/mET30		Keywords/GO	Keywords/GO	Keywords/GO
			Av. Ratio	*T*-test	Av. Ratio	*T*-test	Av. Ratio	*T*-test	Biological process	Cellular component	Molecular function
1105	Aldehyde dehydrogenase family 1 member L1	AL1L1_RAT	0.917	0.528	1.070	0.668	0.741	0.0407	One-carbon metabolism	Cytoplasm	Oxidoreductase
1107	Aldehyde dehydrogenase family 1 member L1	AL1L1_RAT	0.877	0.625	1.080	0.729	0.595	0.0245	One-carbon metabolism	Cytoplasm	Oxidoreductase
1165	rCG56002		0.847	0.46	1.350	0.0618	0.621	0.00384			
1271	Succinate-semialdehyde dehydrogenase_mitochondrial	SSDH_RAT	0.806	0.0326	0.763	0.0404	1.200	0.416	Succinate metabolism, central nervous system	Mitochondrion	Oxidoreductase
1289	Keratin_type I cytoskeletal 18	K1C18_RAT	0.909	0.438	0.438	0.952	0.735	0.05		Cytoplasm, intermediate filament, keratin nucleus	Structural molecule activity
1349	Cystathionine gamma-lyase	CGL_RAT	0.684	0.00202	1.070	0.82	0.820	0.655	Amino acid biosynthesis, cys biosynthesis	Cytoplasm	Lyase
1358	Cystathionine gamma-lyase	CGL_RAT	0.735	0.00429	1.000	0.989	0.820	0.545	Amino acid biosynthesis, Cys biosynthesis	Cytoplasm	Lyase
1395	Serum paraoxonase/arylesterase 1	PON1_RAT	0.980	0.952	0.769	0.0394	1.130	0.305	Multiple regulation/response	Secreted, HDL	Hydrolase

#### Influence of HBCD on liver protein patterns of male rats

Pairwise comparison of mET animals with different exposure to HCBD (0, 3, 30 mg per kg per day) revealed only two proteins with significant changes between 3 and 30 mg per kg per day exposure (Table 2a). In mHT animals 6 proteins were significantly altered, most of them having regulatory or transfer functions (Table 2b). In particular, two of the affected proteins belong to the paraoxonase family (PON1, PON2), a group of enzymes involved in the hydrolysis of organophosphates and lactones. Members of this family differ in cellular localization (PON1 is secreted into circulation, PON2 is present in tissues) and activity, but all act as cellular antioxidants.^23^

In any of these group comparisons, pathway analyses failed to find connections between these few regulated proteins.

#### Influence of thyroid status on liver proteome

This evaluation focused on comparison of male rats of different thyroid status but similar HBCD exposure. Six proteins (8 protein spots) showed statistical differences in pairwise comparison (mHT/mET), but in general only within one of the HBCD dose groups (Table 2c). All 6 proteins were involved in metabolic processes and decreased in hypothyroid status. One of them, serum paraoxonase/arylesterase 1 (PON1), was also differentially abundant in hypothyroid animals as a function of HBCD challenge, at least when comparing responses between 3 and 30 mg per kg per day dose groups.

In summary, only very limited alterations of the liver proteome of male rats were observed between any of the exposed groups and thyroid status. Although a few significant changes of single proteins are noticed, they do not seem functionally connected, belonging to quite different pathways. The only protein family with more than one regulated member in these group comparisons are the PONs. One of their main functions is to metabolize toxic oxidized lipids in LDL (low-density lipoproteins) and HDL (high-density lipoproteins) particles, which may be created by environmental factors or drugs.^24^ The noticed small impacts on PON abundance in our experiments, in parallel to changes seen in leptin concentrations (Table 1), both dependent on HBCD exposure and/or thyroid status, may be interpreted as a small disturbance of lipid metabolism in the liver of male animals.

### Comparison of changes in male and female animals

A previous study from our groups has reported proteomic data as well as hormonal and general animal data in an identical experimental setup with female rats^10^ and performed in parallel. This allows the comparison with the here presented data of males in order to filter for gender specific effects.

General animal and hormone data of animals of both genders are summarized in ESI Table 1† and show the expected differences in body weight, liver weight and gonadotropins between males and females. Especially in ET status, male rats accumulate much less HBCD in adipose tissue compared to female animals (gender effect *P* = 0.001). Van der Ven *et al.*^8^ also reported a lower accumulation of γ-HBCD in liver lipid for mET than for fET rat. However, a major difference between the present study and the investigation by van der Ven and colleagues is that most of their animals received much higher doses of HBCD (up to 200 mg per kg bw per day), and the overall exposure time was 4 times longer compared to the present study. This resulted in changes in liver weights of females, an observation that was not confirmed in our studies, neither for ET nor for HT animals.

#### Comparison of HBCD affected liver proteins of male and female rats

**Fig. 2:**
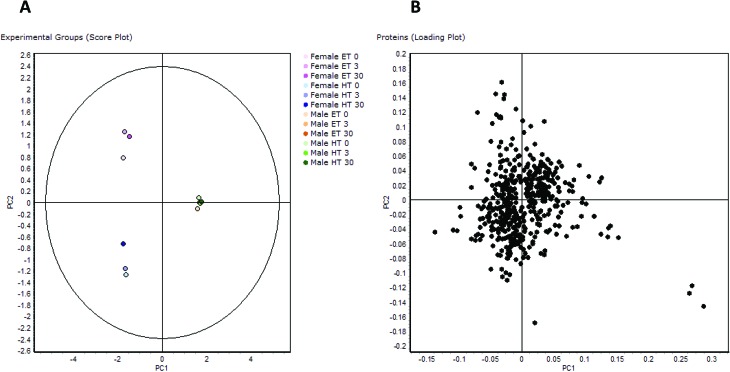
PCA of proteomic data: PCA of the 496 differentially regulated protein spots: (A) comparison of experimental groups (score plot), (B) comparison of all proteins (loading plot).

Protein regulation data of all 12 animal groups (all genders, treatments, thyroid states) were filtered by ANOVA evaluation and resulted in 496 differentially regulated protein spots.^22^ PCA analysis of this spot set showed a clear grouping of values, resulting in three different populations: fET, fHT, and all male animal groups independent of treatment (Fig. 2a). mET and mHT group close together, and there is also no major dispersion in the fET and fHT groups induced by HBCD exposure. Similarly, a heat map and hierarchical clustering based on single spot abundance changes sorted animal groups according to gender and clearly differentiated between thyroid states in females.^22^

The loading plot of the 496 regulated spots visualizes similarity of single spot abundance between PC1 and PC2, and most spots group in a compact cluster (Fig. 2b). The distance of the spots from the centre of the cluster reflects their similarity in regulation behaviour. Some spots/proteins at the border area of the cluster are the ones that had already been found regulated in female rats depending on thyroid status (*e.g.* glutathione-*S*-transferases^10^), but also some typical serum proteins with known functions as protease inhibitors or reactants in inflammation, being known for their concentration differences between genders.^25^ The spots farthest from the centre are those with most different behaviour between PC1 and PC2, *i.e.* three spots in the lower right, belonging to the protein carbonic anhydrase 3. Carbonic anhydrase 3 is known as a growth hormone-dependent liver enzyme, its concentration is gender-dependent, and changed in diabetes/obesity.^26^–^29^ It is one of the proteins already noticed in a previous paper as consistently increased in fHT liver.^10^ Frémont *et al.*^29^ have reported previously the presence of a connection between thyroid status and carbonic anhydrase 3 concentration in skeletal muscle. Gender-dependent abundance differences noticed in this study are even higher, more than 10-fold in mET and more than 5-fold in mHT rats with comparable HBCD exposure, and all highly significant.

Gender-influence in response towards HBCD exposure has been shown on the transcriptomic level, in a study with a much longer exposure time and different doses (most of them much higher than ours), in ET rats.^6^ In the study by Cantón^6^ in females genes related to lipid metabolism, triacylglycerol and cholesterol metabolism were markedly downregulated. In contrast, genes involved in phase I and II metabolism were found upregulated, especially in males, which, for the authors, represented the reason of the lower susceptibility to HBCD of males as compared to females. The described changes were more obvious at higher HBCD doses, and at doses similar to our study, the overall differences between genders and between exposed and unexposed were rather limited. In our study, investigating the proteome, and after the one week exposure, we detected only minor changes in male rats, limited to very few singular proteins. Compared to previous reports, this is a much shorter exposure time, and a time delay between gene regulation and protein synthesis has to be expected. Still, in an identical experiment with females rats^10^ changes in lipid and carbohydrate metabolism were clearly visible, which is in accordance with the transcriptomics results reported by Cantón *et al.*^6^

The only other proteomic study that involved HBCD (administered in a mixture with other flame retardants) and determined its impact on the liver proteome was performed in zebrafish.^30^ Though different to the present study in many main parameters (species, mixed compound, exposure time), it also reported gender-specific effects.

#### Comparison of liver proteins of unexposed male and female rats

**Fig. 3:**
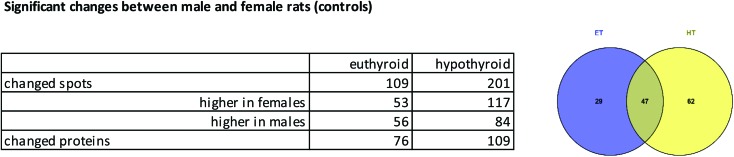
Spot differences between genders: numbers of spots or proteins with altered abundance between male and female controls, as a table and Venn diagram (for proteins).

Gender specific differences in response towards HBCD administration led us to take a closer look at the liver proteome of control rats of both genders and check for gender differences in the 2D-DIGE pattern, by comparing previous data on female animals^10^ with the here presented data for male rats. Unexposed animal groups displayed 109 spots (belonging to 76 proteins) with statistically significant differential abundance between genders for ET, and 201 spots (belonging to 109 proteins) for HT. Changes included an almost similar number of increased and decreased proteins (Fig. 3).

**Fig. 4:**
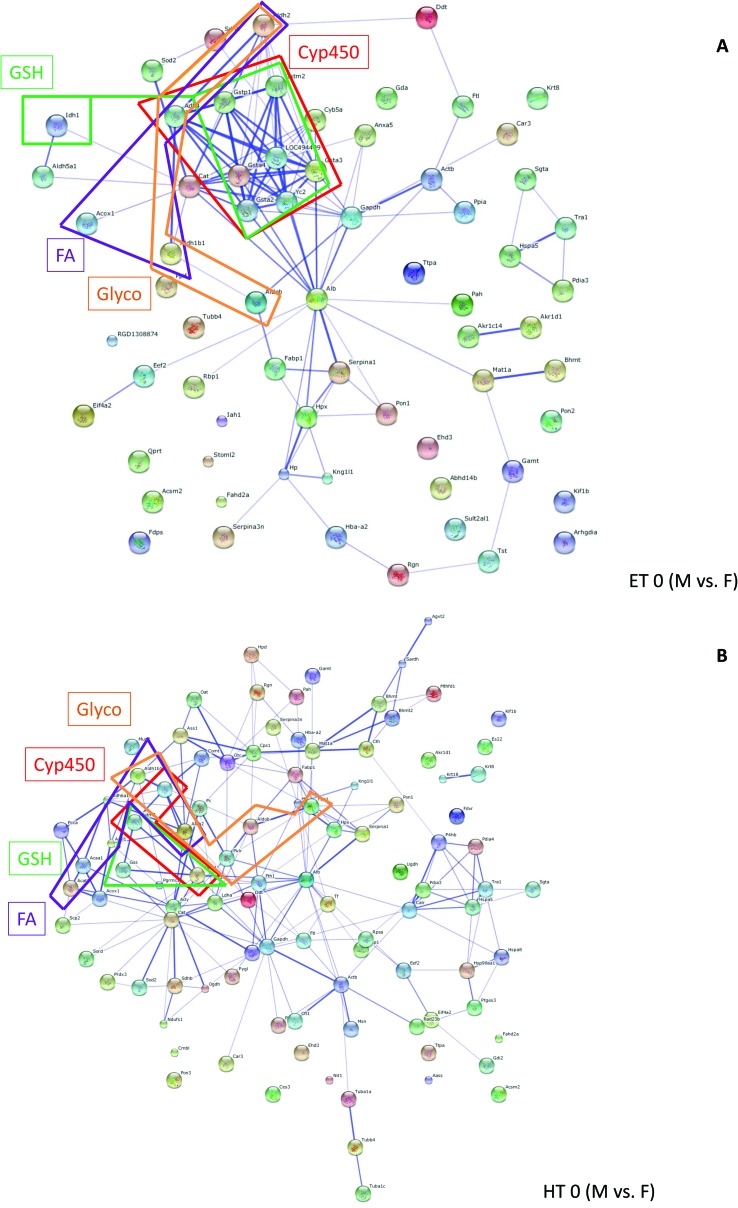
Pathway analysis (STRING): networks created from proteins differentially regulated in male and female rats (controls) in ET animals (A) or HT rats (B). Members from pathways related to Glycolysis/Gluconeogenesis (Glyco), Fatty acid metabolism (FA), Drug metabolism or Metabolism of xenobiotics by cytochrome P450 (CYP), Glutathione metabolism (GSH) are boxed. Boxes are displayed in colour. Further details on pathways and gene/protein names are compiled in ESI Table 3.†

Grouping of these spots and network analysis put the vast majority of these proteins in connection with metabolic processes (ESI Table 3†). For ET animals, there were strong clusters of differentially regulated proteins between genders related to cytochrome P450 (drug metabolism, metabolism of xenobiotics) and glutathione metabolism. Many more members of these protein families were represented in ET animals than in HT animals. In HT animals quite a number of proteins from the fatty acid metabolism were differentially regulated between male and female rats (ESI Table 3,† Fig. 4). Comparison of Fig. 4a and b shows gender differences in the number of interacting proteins, influenced by thyroid status.

Several gender-specific differences in lipid metabolism have been reported earlier, *e.g.* in rats fed a high fat diet different strategies to maintain energetic and metabolic homeostasis in response to feeding have been elicited,^31^,^32^ in the liver proteome,^33^ as well as in plasma proteins^34^ and in a rat model of alcoholic steatohepatitis.^35^ These reports on gender-dependency in lipid metabolism confirm our proteomic results. We noticed small changes in the leptin levels, which were significant in gender*thyroid status interaction, but not in gender effect alone (MANOVA 0.167). Still, gender-related differences in lipid metabolism may also be the reason for the gender differences in HBCD accumulation in adipose tissue in our rats (ESI Table 1†). Studies on liver mitochondrial oxidative metabolism showed gender dimorphism as well,^36^–^38^ namely a different profile of CYP isoforms in microsomes.^7^,^39^,^40^ In our study we could not detect any differentially regulated CYP proteins, probably due to the choice of analysing complete, unfractionated liver lysates, however pathway analysis (Fig. 4, ESI Table 3†) clearly filtered out gender-specific differences in proteins connected to metabolism of xenobiotics/drug metabolism, specifically members of the GST family.

Interestingly, the observed gender-specifically regulated pathways are also the ones affected by HBCD exposure in a gender specific way in the present (and previous) study. Also other reports have shown differences in gender-susceptibility (in rat serum proteins in health and inflammation,^25^,^41^ in mouse proteins in muscle^42^ or in the aging kidney^43^). Our results are thus in line with a recent NIH directive/recommendation for gender-balance in further biomedical research studies.^44^,^45^

## Conclusions

In male rats exposed to levels up to 30 mg per kg HBCD during 7 days, only limited changes in liver protein content and plasma hormone concentrations could be observed. This is in clear contrast to previous findings in female rats^10^ using the same model. Based on pathway analysis involving the whole set of proteins separated and identified by this proteomic study, we could attribute these differences to alterations in metabolism in male and female rats, both under ET and HT conditions, mainly in lipid metabolism and glycolysis/gluconeogenesis, but also in redox and CYP protein related responses. This supports the need to include both genders in animal experiments.

Proteomics proved its usefulness in toxicological investigations, detecting (i) first signs of dysregulation on the protein level after only one week of HBCD exposure, (ii) protein pattern differences in female rats based on thyroid status and – as a main influence – (iii) gender-dependent protein regulation of metabolic pathways, which have a major impact on the body's reactivity towards noxious substances.

## Funding information

This work was supported by contributions from the participating institutions.

## Conflict of interest statement

The authors do not declare any conflict of interest.

## Abbreviations

2D-DIGE: Two-dimensional fluorescence difference gel electrophoresis
CYP: Cytochrome P450
ET: Euthyroid
f: Female
FSH: Follicle-stimulating hormone
GO: Gene ontology
HBCD: Hexabromocyclododecane
HT: Hypothyroid
KEGG: Kyoto Encyclopedia of Genes and Genomes
LC: Liquid chromatography
m: Male
MS: Mass spectrometry
PMF: Peptide mass fingerprint
PON: Paraoxonase/arylesterase
RIA: Radioimmunoassay
STRING: Functional protein association networks
T_3_: Triiodothyronine
T_4_: Thyroxine
TH: Thyroid hormone
TSH: Thyroid stimulating hormone

## Supplementary Material

Supplementary information

Supplementary information

Supplementary information

Supplementary information
