# Supplementary material for: Gender specific differences in the liver proteome of rats exposed to short term and low-concentration hexabromocyclododecane (HBCD)
Source: Toxicol Res (Camb). 2016 Jun 30;5(5):1273–83. doi: 10.1039/c6tx00166a (PMC6062380; doi:10.1039/c6tx00166a)
Supplement: Supplementary file 1 [file TX-005-C6TX00166A-s001.pdf]

Electronic Supplementary Material (ESI) for Toxicology Research.
This journal is © The Royal Society of Chemistry 2016

Data article

Title: ***Dataset of liver proteins of eu- and hypothyroid rats affected in abundance by any of three factors: in vivo exposure to hexabromocyclododecane (HBCD), thyroid status, gender differences***

Authors: Miller, I.[1*], Renaut, J.[2], Cambier, S.[2], Murk, A.J.[3], Gutleb, A.C.[2]†, Serchi, T.[2]†

Affiliations:

[1] Institute for Medical Biochemistry, Department for Biomedical Sciences, University of Veterinary Medicine Vienna, Veterinaerplatz 1, A-1210 Vienna, Austria; e-mail: ingrid.miller@vetmeduni.ac.at

[2] Environmental Research and Innovation (ERIN) Department, Luxembourg Institute of Science and Technology (LIST), 5, avenue des Hauts-Fourneaux, L-4362 Esch-sur-Alzette, Grand-duchy of Luxembourg

[3] Wageningen University, Marine Animal Ecology Group, De Elst 1, 6708 WD Wageningen, The Netherlands.

† Shared Senior Authorship

*** Contact email: ingrid.miller@vetmeduni.ac.at**

Abstract

Effects of exposure of male Wistar rats with different thyroid status (eu-, hypothyroid) to 0, 3 or 30 mg/kg body weight of the flame retardant HBCD for 7 days were studied in comparison with a previous study in females [1]. Proteomic investigation of liver protein patterns obtained by 2D-DIGE was performed and differences between animals groups recorded, based on the factors exposure, thyroid status and gender. All proteins with significantly changed abundance in any of these comparisons were identified by mass spectrometry. General, hormone and proteomic data of both the present and the previous studies are discussed in "Hexabromocyclododecane (HBCD) induced changes in the liver proteome of eu- and hypothyroid female rats" [1] and in "Gender specific differences in the liver proteome of rats exposed to hexabromocyclododecane (HBCD)" [2].

Specifications Table

Subject area	*Biology*
More specific subject area	*Environmental Toxicology*
Type of data	*Tables, figure (PCA), image (annotated gel image)*
How data was acquired	*2D Fluorescence Difference Gel Electrophoresis (2D-DIGE) and mass spectrometry*
Data format	*Analyzed and filtered data*
Experimental factors	*Liver lysates of eu- and hypothyroid male and female rats exposed to different concentrations of HBCD*
Experimental features	*Comparative proteomic analysis of rat liver lysates using 2D-DIGE. Proteins present in differentially abundant protein spots (regarding HBCD exposure, amount, thyroid status, and gender) were identified using MALDI TOF/TOF analysis.*
Data source location	*Origin of samples: Wageningen University, Wageningen, The Netherlands Data collection: Luxembourg Institute of Science and Technology, Esch-sur-Alzette, Luxembourg*
Data accessibility	*Data is with this article as Supplementary Material*

Value of the data

- **Identification of liver proteins from male rats altered due to HBCD exposure**
- **Identification of liver proteins from male rats changed in hypothyroid status**
- **Comparison of male and female rats exposed in a similar exposure experiment, but showing different response in the liver proteome**
- **Identified liver proteins form the basis for a more detailed understanding of involved mechanisms for the investigated compound, of differences in gender susceptibility and of gender dimorphic liver protein composition.**

Data

Rat liver protein lysates show a complex spot pattern in high-resolution two-dimensional electrophoresis (about 3000 spots per gel). Patterns of 24 gels from different exposures of eu- and hypothyroid male rats were evaluated quantitatively. The same number of gels from an identical experiment with female rats from a previously described study [1, 3] was re-evaluated together with the newly obtained males' data, and additional MS identifications of regulated spots in females vs. males were performed. Data from different animals groups in both genders were compared, taking different aspects into account (HBCD exposure, thyroid status, gender). Statistically significant fold-changes of at least 30 % between groups (P<0.05 within group) were considered to be relevant.

Suppl. Figure 1 presents the master gel of the combined gel set, and all spots with significant abundance changes in any of the performed comparisons are labelled. Overall, single proteins in 496 spots, selected

by ANOVA evaluation, were successfully identified by MS analyses. Their spot numbers together with relevant peptide identifications are listed in **Suppl. Table 1**. Abundance changes of the various animal groups are compiled in **Suppl. Table 2**. Hierarchical clustering gave a clear grouping of regulated spots according to gender, and in females also according to thyroid state (**Suppl. Figure 2**). This is in line with PCA results (score plot of experimental groups) reported in [2]. The loading plot of this PCA is shown in more detail in **Figure 1**, with additional identification of single proteins in the border area or close to the main cluster.

Experimental Design, Materials and Methods

Animals, treatment and experimental protocol

The animal experiment was detailed in [1, 2] and approved under number 2006-051 by the Animal Welfare Committee of Wageningen University. In brief, male Wistar WU (HsdCpbWU) rats with normal or reduced thyroid function (hypothyroid) were orally exposed to 0, 3 or 30 mg/kg bw / d HBCD, respectively, for 7 consecutive days. Four liver samples per group were analyzed by proteomic methods. The experimental setup was identical to the one used in [1], but applying it on male rats.

Proteomic Analysis

Rat liver lysates were subjected to two-dimensional fluorescence difference gel electrophoresis (2D-DIGE) as previously described, without modifications, to make it compatible to the previously performed study in females [1-3]. This comprised a classical 2D-DIGE experiment, by separating CyDye-labeled proteins from liver lysates in a non-linear 3-10 pH-range and subsequently in large-format SDS-PAGE gels (260 x 200 x 1 mm). Gel images (acquired on a Typhoon 9400) were evaluated with the DeCyder 7.0 software package (both GE Healthcare, Diegem, Belgium), including all gels of the previous study on females in matching and statistics [1, 3]. Univariate and multivariate analyses were applied to highlight differentially regulated spots (fold change at least 1.3) with a *P*-value in the respective univariate ANOVA or two way ANOVA <0.05.

Spots differentially regulated in any of the group comparisons (including samples/gels from males and females) were automatically picked, trypsin digested and proteins identified by MALDI-TOF/TOF 5800 (Sciex) as previously described [1-3]. This included also spots from female rat livers previously not

analyzed, as only now they were found regulated in the comparison with male rats' data. Protein identification was based on searches of mass fingerprints (PMF) and MS/MS spectra against the SwissProt database with "*Rattus norvegicus*" as taxonomy, using the ProteinPilot software (Sciex, Nieuwerkerk aan den Ijssel, The Netherlands) and the searching algorithm MASCOT (Matrix Science, www.matrixscience.com, London, UK). For each spot one protein mass fingerprint and up to 8 MS/MS spectra were generated. Parameters for the search were set as in the previous study [1, 3]: up to two missed cleavages allowed, 100 ppm tolerance in PMF, 0.75 Da mass tolerance for precursor ion mass, carbamidomethyl cysteine as fixed modification, oxidation of methionine and oxidation of tryptophan (single oxidation, double oxidation and kynurenin) as variable modifications. Identifications were considered to be significant when the combined MOWSE score had $P<0.05$.

For statistics the Extended Data Analysis (EDA) module as well as univariate analysis (ANOVA and t-test) and multivariate analysis (two way ANOVA), all part of the Decyder 7.0 software package, were applied.

References

[1] Miller, I, Serchi, T, Cambier, S, Diepenbroek, C, Renaut, J, Van den Berg, JHJ, Kwadijk, C, Gutleb, AC, Rijntjes, E, Murk, AJ. (2016). Hexabromocyclododecane (HBCD) induced changes in the liver proteome of eu- and hypothyroid female rats, *Toxicol Lett* **245**, 40-51. http://dx.doi.org/10.1016/j.toxlet.2016.01.002.

[2] Miller, I, Diepenbroek, C, Rijntjes, E, Renaut, J, Swarts, H, Kwadijk, C, Cambier, S, Murk, AJ, Gutleb, AC, Serchi, T. Gender specific differences in the liver proteome of rats exposed to short term and low-concentration hexabromocyclododecane (HBCD), *Toxicol Res*, submitted in parallel.

[3] Miller, I, Serchi, T, Cambier, S, Diepenbroek, C, Renaut, J, Van den Berg, JHJ, Kwadijk, C, Gutleb, AC, Rijntjes, E, Murk, AJ. (2016). Dataset of liver proteins of eu- and hypothyroid female rats changing abundance upon in vivo exposure to hexabromocyclododecane (HBCD), *Data in Brief* **7**, 386-392. http://dx.doi.org/10.1016/j.dib.2016.02.047.

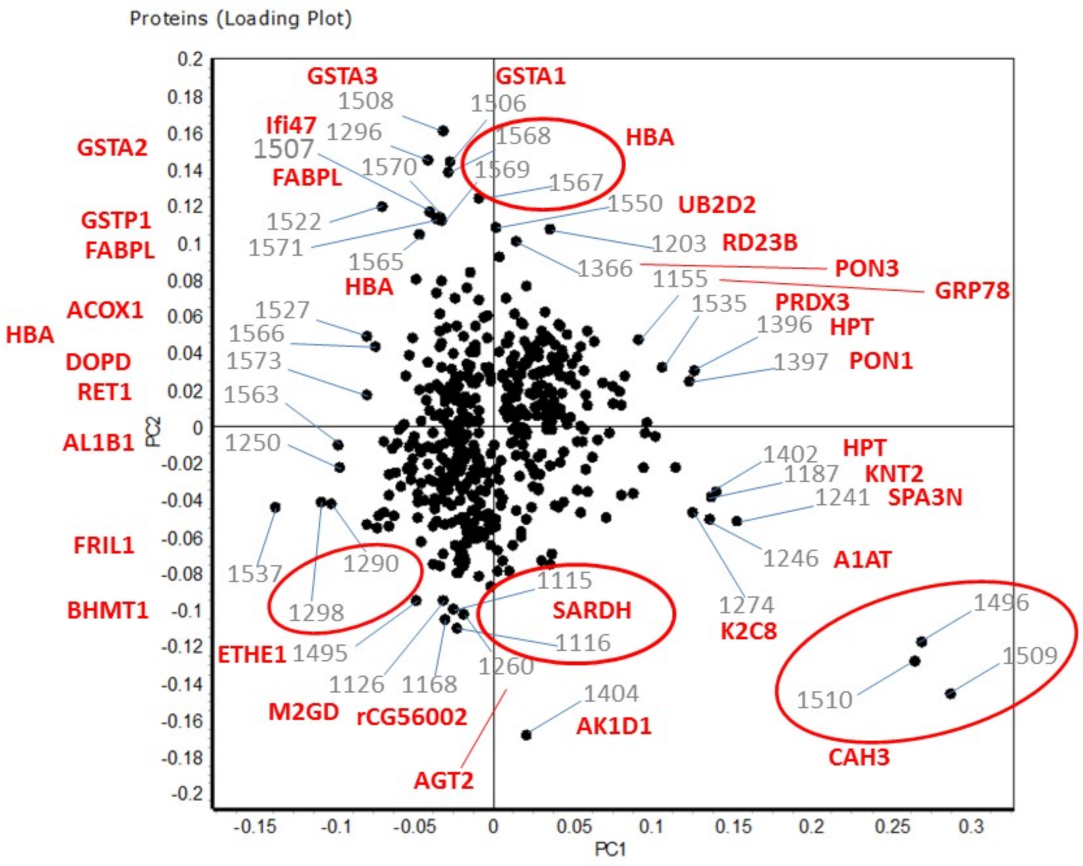

Figure 1:

PCA Loading plot of the 496 differentially regulated spots. Spot numbers (corresponding to labelling in gel image in **Suppl. Figure 1** and regulation data in **Suppl. Table 2**) and UniProt accession numbers (**Suppl. Table 1**) used for labelling.

Abbreviations:

A1AT_RAT, Alpha-1-antiproteinase

ACOX1_RAT, Peroxisomal acyl-coenzyme A oxidase 1

AGT2_RAT, Alanine--glyoxylate aminotransferase 2_mitochondrial

AK1D1_RAT, 3-oxo-5-beta-steroid 4-dehydrogenase

AL1B1_RAT, Aldehyde dehydrogenase X_mitochondrial

BHMT1_RAT, Betaine--homocysteine S-methyltransferase 1

CAH3_RAT, Carbonic anhydrase 3

DOPD_RAT, D-dopachrome decarboxylase

protein ETHE1_mitochondrial

FABPL_RAT, Fatty acid-binding protein_liver

FRIL1_RAT, Ferritin light chain 1

GRP78_RAT, 78 kDa glucose-regulated protein

GSTA1_RAT, Glutathione S-transferase alpha-1

GSTA2_RAT, Glutathione S-transferase alpha-2
GSTA3_RAT, Glutathione S-transferase alpha-3
GSTP1_RAT, Glutathione S-transferase P
HBA_RAT, Hemoglobin subunit alpha-1/2
HPT_RAT, Haptoglobin
Ifi47 protein
K2C8_RAT, Keratin_type II cytoskeletal 8
KNT2_RAT, T-kininogen 2
M2GD_RAT, Dimethylglycine dehydrogenase_mitochondrial
PON1_RAT, Serum paraoxonase/arylesterase 1
PON3_RAT, Serum paraoxonase/lactonase 3
PRDX3_RAT, Thioredoxin-dependent peroxide reductase_mitochondrial
rCG56002
RD23B_RAT, UV excision repair protein RAD23 homolog B
RET1_RAT, Retinol-binding protein 1
SARDH_RAT, Sarcosine dehydrogenase_mitochondrial
SPA3N_RAT, Serine protease inhibitor A3N
UB2D2_RAT, Ubiquitin-conjugating enzyme E2 D2

Supplemental material:

Suppl. Figure 1: Image of a rat liver 2D-DIGE gel (master gel, grey level image). All spots with statistically significant abundance changes in any of the different comparisons (HBCD exposure, thyroid state, gender) are labelled; spot numbers refer to identifications in **Suppl. Table 1**. For data on protein abundance, see **Suppl. Table 2**.

Suppl. Figure 2: Heat map and hierarchical clustering of all 496 differentially regulated spots.

Suppl. Table 1: Protein identifications by MALDI-TOF/TOF analysis.
For spot locations on the gel see gel image in **Suppl. Figure 1**.

Suppl. Table 2: All spots found affected in the complete/combined dataset (male and female rats). Ratios between different animal groups (n=4). Abbreviations: f, females; m, males; ET, euthyroid rats; HT, hypothyroid rats; 0, 3, 30 refer to amounts of HBCD (mg/kg bw / d) administered in the 7-day exposure period.
