# Supplementary material for: Gender specific differences in the liver proteome of rats exposed to short term and low-concentration hexabromocyclododecane (HBCD)
Source: Toxicol Res (Camb). 2016 Jun 30;5(5):1273–83. doi: 10.1039/c6tx00166a (PMC6062380; doi:10.1039/c6tx00166a)
Supplement: Supplementary file 2 [file TX-005-C6TX00166A-s002.pdf]

Electronic Supplementary Material (ESI) for Toxicology Research.
This journal is © The Royal Society of Chemistry 2016

Suppl. Figure 1: Image of a rat liver 2D-DIGE gel (master gel, grey level image). All spots with statistically significant abundance changes in any of the different comparisons (HBCD exposure, thyroid state, gender) are labelled ; spot numbers refer to identifications in **Suppl. Table 1**. For data on protein abundance, see **Suppl. Table 2**.

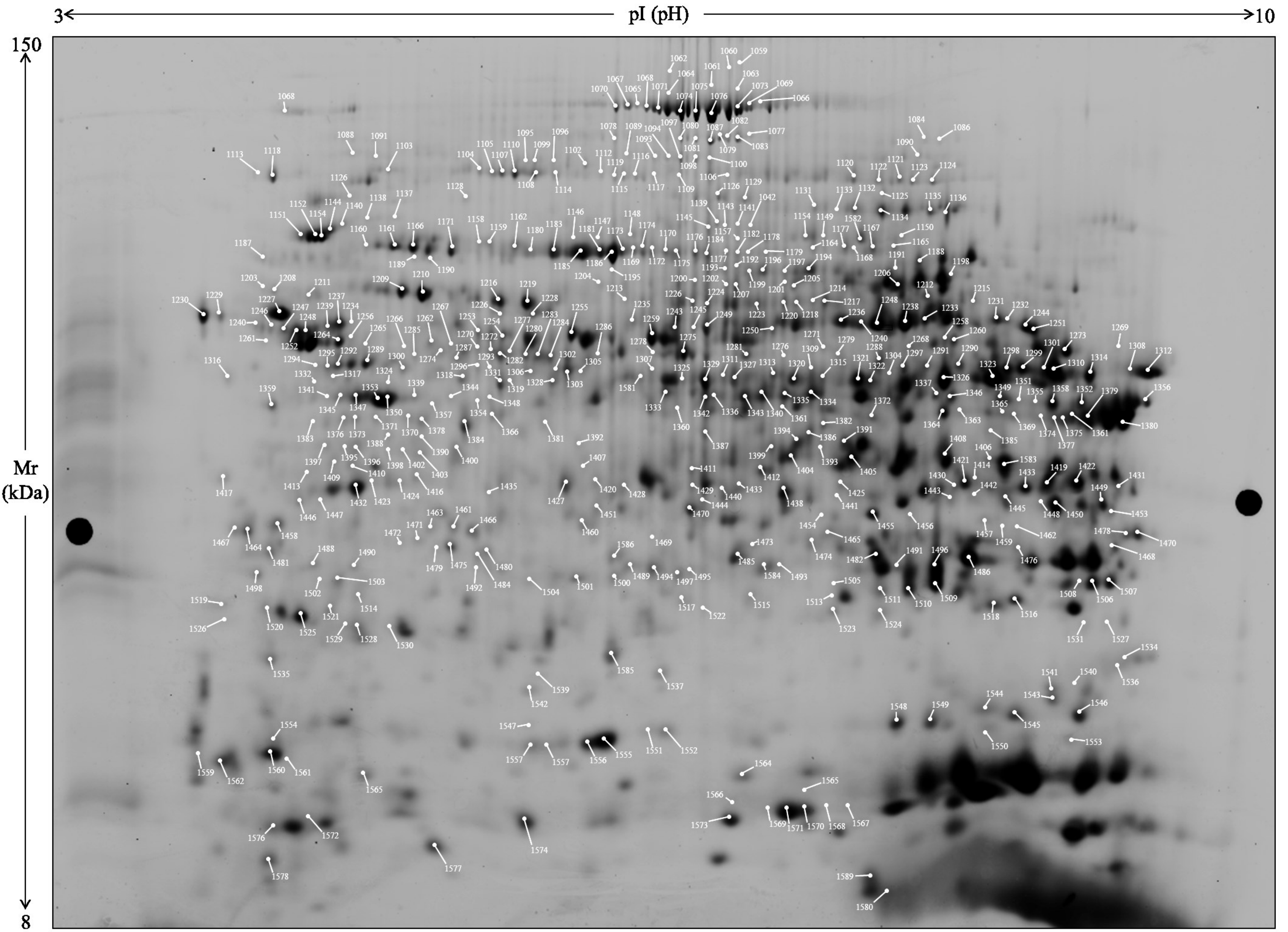

3
pI (pH)
10
150
Mr
(kDa)
8

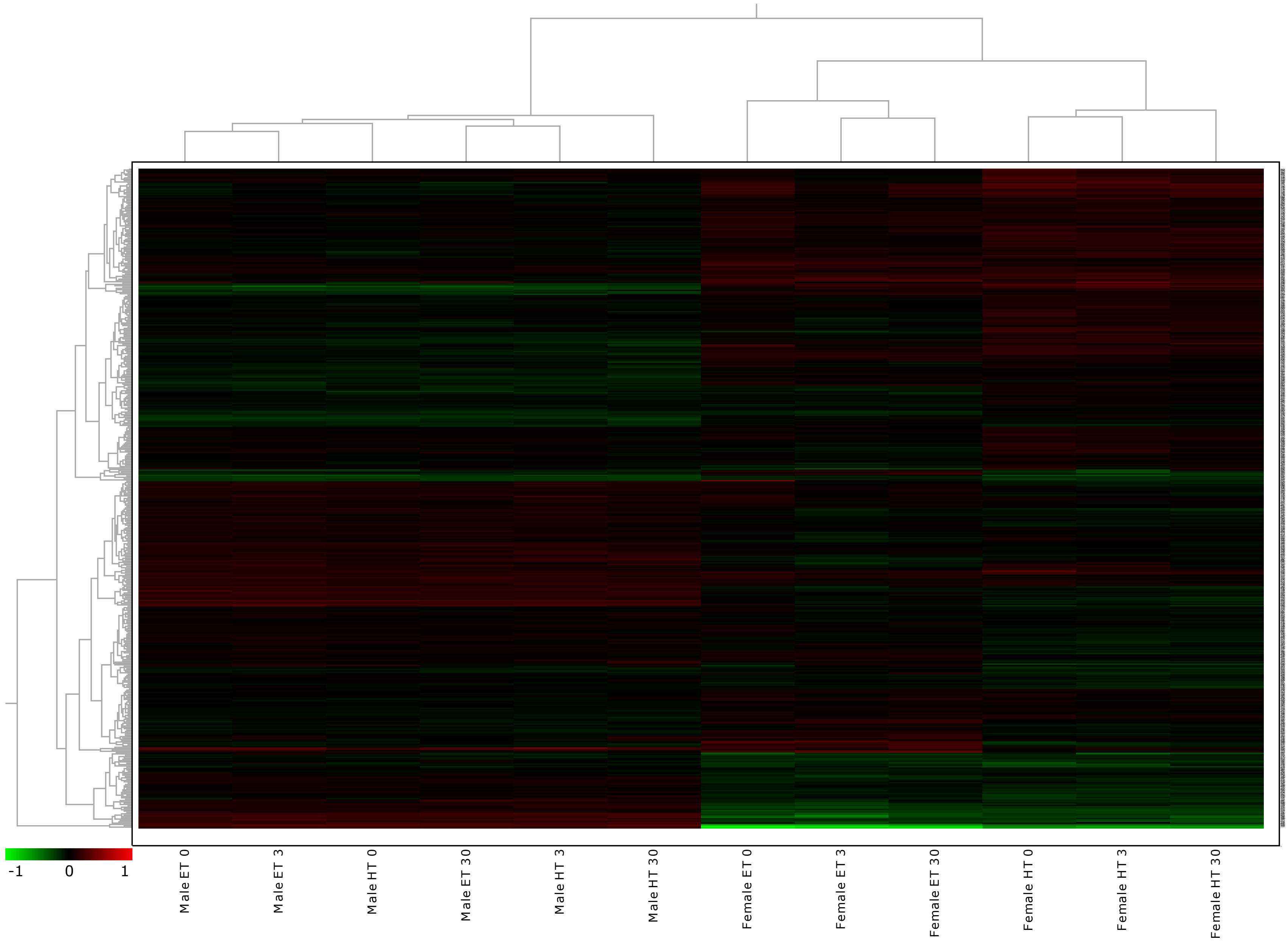

Suppl. Figure 2: Heat map and hierarchical clustering of all 496 differentially regulated spots.
